# Ag/Ag_3_PO_4_ Nanoparticle-Decorated Hydroxyapatite Functionalized Calcium Carbonate: Ultrasound-Assisted Sustainable Synthesis, Characterization, and Antimicrobial Activity

**DOI:** 10.3390/ma16041338

**Published:** 2023-02-04

**Authors:** Alessandro Di Michele, Morena Nocchetti, Donatella Pietrella, Loredana Latterini, Giulia Quaglia, Ilaria Mattu, Giuseppina Padeletti, Saulius Kaciulis, Eleonora Bolli, Valeria Ambrogi

**Affiliations:** 1 Dipartimento di Fisica e Geologia, University of Perugia, Via Alessandro Pascoli, 06123 Perugia, Italy; 2 Dipartimento di Scienze Farmaceutiche, University of Perugia, Via del Liceo 1, 06123 Perugia, Italy; 3 Dipartimento di Medicina e Chirurgia, University of Perugia, Via Gambuli, 1, 06132 Perugia, Italy; 4 Nano4Light Lab, Dipartimento di Chimica, Biologia e Biotecnologie, University of Perugia, Via Elce di Sotto 8, 06123 Perugia, Italy; 5 Institute for the Study of Nanostructured Materials, ISMN-CNR, Via Salaria Km 29,300, 00015 Rome, Italy

**Keywords:** ultrasound-assisted sustainable synthesis, hydroxyapatite functionalized CaCO_3_, Ag/Ag_3_PO_4_ nanoparticles, antibiofilm property, antibacterial activity, cytotoxicity evaluation

## Abstract

Silver nanoparticles are usually prepared by the reduction of silver cations through chemical and non-sustainable procedures that involve the use of reducing chemical agents. Therefore, many efforts have been made in the search for sustainable alternative methods. Among them, an ultrasound-assisted procedure could be a suitable and sustainable method to afford well-dispersed and nanometric silver particles. This paper describes a sustainable, ultrasound-assisted method using citrate as a reducing agent to prepare silver@hydroxyapatite functionalized calcium carbonate composites. For comparison, an ultrasound-assisted reduction was performed in the presence of NaBH_4_. The composites obtained in the presence of these two different reducing agents were compared in terms of nanoparticle nature, antimicrobial activity, and cytotoxic activity. The nanoparticle nature was investigated by several techniques, including X-ray powder diffraction, field-emission scanning electron microscopy, transmission electron microscopy, UV–Vis spectroscopic measurements, and X-ray photoemission spectroscopy. Nanoparticles with a predominance of Ag or Ag_3_PO_4_ were obtained according to the type of reducing agent used. All composites were tested for antimicrobial and antibiofilm activities against Gram-positive and Gram-negative (*Staphylococcus aureus* and *Pseudomonas aeruginosa*, respectively) bacteria and for cytotoxicity towards human skin keratinocytes and human fibroblasts. The nature of the nanoparticles, Ag or Ag_3_PO_4_, and their predominance seemed to affect the in vitro silver release and the antimicrobial and antibiofilm activities. The composites obtained by the citrate-assisted reduction gave rise to the best results.

## 1. Introduction

For many years, compounds containing ionic or metallic silver have been known for their antibacterial and antifungal properties and have been applied in many fields. More recently, thanks to the establishment of nanotechnological methods, silver nanostructures have further increased their application perspectives because of their improved interface properties and their enormous increase in surface/volume ratios with the reduction in their size. Thus, silver nanoparticles stand out for their contributions in many applications, such as drug delivery [1], wound dressings [2,3], nanomedicine [4], dentistry [5], chemical sensing [6], data storage [7], cell biology [8], the cosmetic [9] and textile industries [10], and food packaging [11].

Nanoparticles can be obtained by two different procedures, such as the top-down and bottom-up approaches. Among the bottom-up procedures, the reduction of silver cations through physical [12,13] or chemical procedures [14] is usually the most used process. The chemical reduction preparations involve the use of reducing chemical agents [15,16,17] in different media and a capping agent capable of controlling the nanoparticle size. The most commonly used reducing agents are sodium citrate, poly-ethylene glycol block copolymers, ascorbate, Tollen’s reagent, sodium borohydride, and N,N-dimethylformamide [18,19,20]. Most of these agents are toxic or lead to non-ecofriendly byproducts. Among them, nowadays, the use of citrate, which has the double function of a reducing agent and a capping agent, has been established for its safety and biocompatibility. Recently, Ag/graphene composites were obtained by green synthesis-reducing silver acetate in water with trisodium citrate and high-power ultrasound waves [21]. The fabrication of nanocomposites using sustainable ultrasound-assisted methods offers several advantages, including increased reaction rate and yield, improved nanoparticle quality with regard to regular size distribution and spherical shape [22,23], and prevention of particle agglomeration [24,25]. Moreover, the ultrasound-assisted method is an emerging technology in the following areas: (i) the fabrication of nanocomposites for drug delivery [26] and scaffolds for gingival cell growth [27]; (ii) environmental processes for wastewater treatment [28]; (iii) the extraction of functional compounds from foods [29] and dairy product processing [30]; (iv) gas production, such as H_2_ [31]. The effect of ultrasound is due to a physical phenomenon called acoustic cavitation. When ultrasound waves propagate in a fluid with dissolved gases, it causes the formation of bubbles, their growth, and implosive collapse. This phenomenon can also induce a shock wave in the solution and lead to the quick impact of the liquid on the surface of the particles, which promotes the formation of non-agglomerated nanocomposites [32]. In comparison to traditional procedures, the main advantages of ultrasound use are shorter reaction times, milder conditions, increased yields, and the use of water as a solvent [33].

Recently, inorganic microparticles constituted of hydroxyapatite (HA) and calcium carbonate have gained attention by proving to have a large number of new applications. They have been evaluated as excipients in drug formulations because of their good stability and slow biodegradability; moreover, they can be easily produced and are biocompatible [34]. Hydroxyapatite/calcium carbonate composite microspheres have also been proposed for applications in bone tissue engineering [35,36]. In fact, their chemical compositions are similar to the inorganic phase of teeth and bones [37], and this feature suggests the possibility of using these materials as implants in bone surgeries with some advantages in comparison to pure HA. In fact, the presence of calcium carbonate, which is more soluble, could modify the slow degradation of HA, making it faster and allowing for implant bone resorption [36,38,39].

Implant-associated infections are a significant clinical problem and have a huge impact regarding morbidity, mortality, and medical public costs [40]. If an implant infection occurs, long-term antibiotic therapy, which is systemically administered, is usually necessary but not always with a positive onset because of the diffusion of antibiotic resistance. In the cases of more severe infections, invasive surgery is required for surgical debridement of the affected tissue, and, in the worst cases, the complete removal of the implant could be necessary. One of the proposed strategies to prevent this problem is the use of composites of HA and silver [41,42,43,44,45,46,47,48]. The main methods proposed to obtain HA and silver biomaterials were reviewed by Guo et al. [49]. They consist of doping HA with silver during its preparation [50,51,52,53], the adsorption or deposition of silver nanoparticles that have been previously obtained by reduction on HA [41,44,45,54,55,56], and contact between HA and silver salt without chemical reducing agents [46,57]. In this late method, Ag/Ag_3_PO_4_ nanoparticles were formed. Gottardo et al. obtained Ag/HA nanocomposites by a one-pot microwave-assisted solvothermal method [43], whereas Calabrese et al. obtained the direct growth of Ag nanoparticles on an HA surface by a green photochemical synthesis [47]. Most of the above methods involve the use of a chemical reductant and other reagents and/or unsustainable reaction conditions (high temperatures for a long time), and, thus, an ultrasound-assisted procedure may have advantages over traditionally employed methods, mainly in terms of sustainability. In the above-cited papers, silver–HA composites show activity against both Gram-positive and Gram-negative bacteria and against yeasts. This activity is also present at low silver concentrations and improves as the silver content increases. Silva et al. [41] and Ambrogi et al. [58] reported a literature review of research on the antimicrobial activity of silver composites.

Previously, a hydroxyapatite functionalized calcium carbonate, named OMP, which is characterized by a highly developed surface area and porous structures, was used for obtaining silver@composites. The nature of the silver in the prepared composites was deeply investigated by XPS [57], showing that the oxidation state of the silver depends on the synthetic procedures used. The composites obtained without reducing agents were composed mainly of Ag_3_PO_4_ nanoparticles and showed good antimicrobial activity [58].

In this paper, silver@composites were obtained by a sustainable approach called ultrasound-assisted citrate reduction. The silver nature and morphology were investigated, and the antimicrobial and cytotoxic activities of the composites were evaluated. For comparison, these composites were compared with those obtained by an analogous, ultrasound-assisted, non-sustainable procedure with NaBH_4_.

## 2. Materials and Methods

### 2.1. Materials

The hydroxyapatite functionalized calcium carbonate (OMP) was kindly offered by Omya Italia (Avenza (MS)-Massa, Carrara, Italy). Sodium citrate, silver acetate, and sodium borohydride were acquired from Alfa Aesar (Karlsruhe, Germany). The other reagents and solvents were of reagent grade. Deionized water, obtained by reverse osmosis (MilliQ system, Millipore, Rome, Italy), was used.

### 2.2. Ultrasound-Assisted Synthesis of Silver Nanoparticles on OMP

Four OMP@silver composites were prepared by sonochemical methods. In each synthesis, 1 g of OMP was dispersed in 60 mL of a silver acetate aqueous solution, and 50 mL of a reducing agent solution (sodium citrate or NaBH_4_, generically indicated as RA) was successively added dropwise under ultrasound irradiation (for 30 min and at 50% of amplitude). The reaction conditions, the concentrations of the reactants, and the Ag/RA molar ratios are reported in Table 1.

The ultrasound irradiation was generated using the ultrasonic processor VCX750 (Sonics & Materials, Inc., Newton, CT, USA), operating at 20 kHz and having a 13 mm diameter tip.

After centrifugation of the dispersion (at 6000 rpm for 10 min), the recovered solid was washed three times with ethanol and finally dried in an oven at 37 °C for 24 h. The composites were stored under P_2_O_5_. As a comparison, two composites were synthesized without ultrasound irradiation. 

### 2.3. Characterization

The XRPD patterns were recorded with a diffractometer in Bragg–Brentano geometry (Bruker D8 Advance, Bruker AXS GmbH, Karlsruhe, Germany), provided with an Lynxeye XE-T fast detector and CuKα radiation (operative conditions: 40 kV and 40 mA, a step size of 0.033° 2θ, and a step scan of 30 s). The Bruker DIFFRAC.EVA V5 software equipped with the COD database was used for the phase identification.

The metal contents were determined using inductively coupled plasma optical emission spectrometers (ICP-OES) (Varian 700-ES series, Agilent Technologies, Mulgrave, Victoria, Australia). A known sample amount was solubilized in concentrated HNO_3_, and the solution was properly diluted in deionized water and, finally, analyzed.

UV–Vis spectra, used to investigate the optical properties of the composites, were collected by a Varian (Cary 4000) spectrophotometer (Palo Alto, CA, USA), provided with a 150 mm integration sphere, and a barium sulfate tablet was used as a reference.

The composite morphologies were investigated by a transmission electron microscope (TEM) with a Philips 208 (FEI, Hillsboro, OR, USA), operating at an accelerating voltage of 100 kV. The samples were prepared by dropping a composite ethanolic dispersion on a copper grid precoated with a Formvar film. The solvent was allowed to evaporate in the air at room temperature before the analysis. 

The composite morphologies were also investigated by field-emission scanning electron microscopy (FE-SEM) through the LEO 1525 ZEISS instrument (Jena, Germany), equipped with Inlens and angle-selective back-scattered (AsB) detectors. A Bruker Quantax EDX was used for the elemental composition and chemical mapping. The composites were metallized by sputtering with chromium (8 nm) after their deposition on conductive carbon adhesive tape.

X-ray photoemission spectra (XPS) were collected by the Escalab MkII electronic spectrometer (VG Scientific Ltd., East Grinstead, UK). The composites were deposited on pure Au foil (99.99%). The analyses were performed using a chamber base pressure of about 2 × 10^−9^ mbar and an unmonochromatized Al Kα source. The photoemission spectra were registered by a 5-channeltron detection system, and, during their acquisition, the analyzer was set to a constant pass energy of 40 eV. The Auger spectra were collected at a pass energy of 100 eV to increase the signal-to-noise ratio. The analyzer input slits were set to large area mode (the diameter of the analyzed area was about 10 mm).

The Avantage v.5 software (Thermo Fisher Scientific Ltd., East Grinstead, UK) was applied for the experimental data acquisition and processing [57].

### 2.4. Silver Release

The samples (10 mg) were dispersed in 10 mL of deionized water at 37 °C by stirring (60 rpm). Withdrawals of the release fluid (4 mL) were performed at defined time intervals, and the silver content was measured through ICP-OES after the addition of 1 mL of concentrated HNO_3_ solution. Every withdrawal was replenished with deionized water (4 mL) that had previously been heated to 37 °C ± 0.5 °C. The experiment was repeated three times, and the reported data were averaged.

### 2.5. Bacteria

The Gram-positive *Staphylococcus aureus* (ATCC 29213) and the Gram-negative *Pseudomonas aeruginosa* (ATCC15692) bacteria, which were maintained on Muller Hinton agar (MHA), were used as the model strains. Before the tests, one colony was inoculated in Muller Hinton broth (MHB) and incubated for 24 h at 37 °C.

### 2.6. In Vitro Susceptibility Test

The minimum inhibitory concentration (MIC) was evaluated, following the CLSI (Clinical Laboratory Standards Institute) standards (CLSI, 2015), using gentamicin as a control and 96-well U-bottom microdilution plates. The composites and gentamicin were dispersed in MHB at a concentration of 1000 µg/L. Serial dilutions of 1:2 in MHB were performed, and 100 μL of the diluted dispersion was placed in a 96-well culture plate. A volume of 100 μL of the bacterial suspension was diluted in MHB to a concentration of 10^4^–10^5^ microorganisms/mL and added to each well. The microplates were incubated for 24 h at 37 °C. Each experiment was performed at least three times. The positive growth control was composed of untreated bacteria. The MIC was defined as the lowest concentration that inhibited the bacteria visible growth.

### 2.7. Biofilm Inhibition Assay

The static biofilm inhibition of the composites was evaluated as reported in reference [59], with some modifications. The cultures of *S. aureus* or *P. aeruginosa* were diluted at a ratio of 1:100 into 15 mL of 2% sucrose-supplemented MHB in the presence or absence of gentamicin, which was used as a positive control, and of the composites tested at different concentrations (10, 100, and 1000 µg/L). The cultures were seeded in 96-well microtiter plates and incubated at 37 °C for 24 h. After the incubation, the biofilm grown in each well was washed with deionized water (2 × 200 μL). The dried biofilm of each well was treated with 100 μL of 0.4% crystal violet for 30–45 min. The wells were washed with deionized water (4 × 200 μL) and then discolored with 200 μL of 95% ethanol for 45 min. An aliquot of the discolored solution (100 μL) was poured into the well of a new plate, and the absorbance of the solution was measured at 570 nm in a microplate reader (Infinite M200 pro, TECAN, Mannedorf, Switzerland). The comparison of the absorbance values of the compound-treated wells versus the untreated control wells allows for the determination of the amount of formed biofilm. The test was performed at least in triplicate for each composite concentration.

A fluorescence assay was performed to evaluate the live cells in the biofilm. The floating cells of the *S. aureus* or *P. aeruginosa* biofilms after 24 h of incubation were withdrawn and stained with the LIVE/DEAD BacLight Bacterial Viability Kit (ThermoFisher Scientific, Milan, Italy). A total of 100 μL of this solution was poured in the wells and incubated at room temperature for 15 min. After removing the excess solution, the biofilm was washed and analyzed by a fluorimeter. The live cells were determined at an excitation wavelength (λ_exc_) of 485 nm and an emission wavelength (λ_em_) of 530 nm, while the dead cells were determined at λ_exc_ of 485 nm and λ_em_ of 630 nm. The live/dead cell ratio was calculated and reported.

### 2.8. Cell Lines

NCTC2544 (human skin keratinocytes; Istituto Nazionale per la Ricerca sul Cancro, HL97002) and HuDe (human dermal fibroblast cell line; Istituto Zooprofilattico Sperimentale della Lombardia e dell’Emilia Romagna, BS PRC 41) cells were used. The culture medium was composed of RPMI 1640 with 10% FBS (fetal bovine serum), 2 mM of glutamine, 100 μg of streptomycin/mL, and 100 U (units) of penicillin (cRPMI). The confluent cultures were split using 0.25% trypsin/EDTA. The monolayers were incubated until cell detachment at room temperature for 5–10 min. The cell dispersions were successively obtained by adding fresh medium, and the suspension was centrifuged and properly diluted.

### 2.9. Cytotoxicity Evaluation

The cytotoxicity of the composites was evaluated by determining the cell ATP level using the ViaLight^®^ Plus Kit (Lonza, Basel, Switzerland). ATP, which is present in metabolically active cells, can be measured by a bioluminescent method that utilizes the enzyme luciferase.

The cell dispersions with a concentration of 2 × 10^5^/mL were seeded in a flat-bottom 96-well culture plate and incubated to obtain the cell monolayer formation. The composites were dispersed in cRPMI at a concentration of 1000 µg/L. Serial dilutions at 1:2 in cRPMI were performed to obtain different concentrations (from 1000 μg/mL to 0.5 μg/mL). The dispersions of the composites were added to the cell monolayer, and the plates were incubated at 37 °C for 4 or 24 h. The control was composed of the untreated cells, and each dispersion was tested three times. The ATP was extracted from the cells after the incubation with a cell lysis reagent. The extracted ATP was left to react with AMR Plus (ATP Monitoring Reagent Plus) for 2 min, and the luminescence was measured by a microplate luminometer (TECAN). The results are reported as the concentration required to reduce the cell viability by 50% (CC_50_) compared to the untreated controls.

## 3. Result and Discussion

### 3.1. Composite Characterization

**Figure 1:**
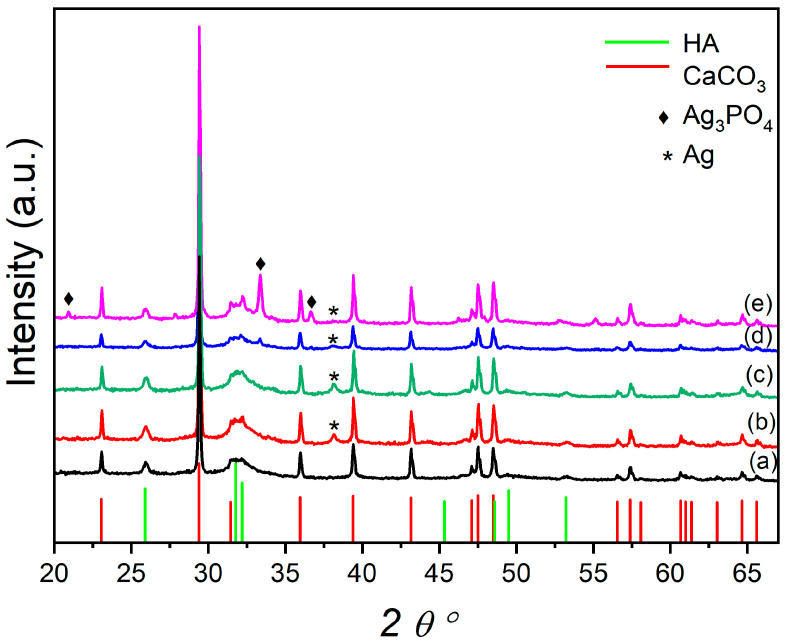
XRD pattern of OMP (**a**) compared with the OMP@silver composites: US50B (**b**), US100B (**c**), US50C (**d**), and US100C (**e**). Pattern lines: CaCO_3_ (COD ID: 9015390), red lines; HA (COD ID: 9011097), green lines; Ag_3_PO_4_ (COD ID: 2106404), ◆; Ag (COD ID: 9008459) *.

Figure 1 reports the XRPD of the plain OMP and the OMP@silver composites. The OMP sample exhibits typical reflections of CaCO_3_ and hydroxyapatite phases; additional phases are formed in the OMP@silver composites prepared using the reducing agents, NaBH_4_ (US50B and US100B) and trisodium citrate (US50C and US100C). The US50B and US100B spectra show a reflection at the 38.12 2 theta degree ascribable to the (111) crystalline planes of the cubic metallic Ag (reference pattern COD number: 9008459) (Figure 1b,c). This reflection is also detectable in the US50C and US100C, even if it has a very small intensity and is very broad. However, in these latter samples, the formation of cubic Ag_3_PO_4_ (reference pattern COD number: 2106404) is clearly visible (Figure 1d,e) and increases with the amount of silver acetate used in the synthesis from US50C to US100C. In the employed synthetic procedure, the predominance of the Ag or Ag_3_PO_4_ nanoparticles in the OMP@silver composites can be explained considering that silver may undergo the following two processes: the reduction to metallic silver or the formation of silver phosphate. In the presence of NaBH_4_, due to its high reduction power and high concentration, the reduction in both composites is dominant. Conversely, when milder reducing conditions are used with the citrate, the formation of Ag_3_PO_4_ can be competitive, and the amount of Ag_3_PO_4_ nanoparticles increases as the silver acetate concentration is doubled (US100C).

The dimensions of the silver and Ag_3_PO_4_ nanoparticles were obtained by the Scherrer equation and are reported in Table 2, along with the total silver loading obtained by ICP.

**Figure 2:**
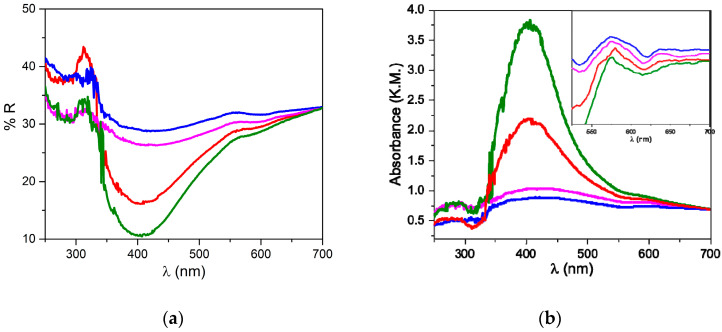
UV–Vis reflectance (**a**) and absorption (**b**) spectra (in Kubelka–Munk units) of US50B (red line), US100B (green line), US50C (blue line), and US100C (pink line); Inset first derivative of the absorption spectrum.

UV–Vis spectra were collected in order to obtain information on the nature and size of the silver nanoparticles. Figure 2a presents the reflectance spectra of the composites, from which their absorption profile was determined using the Kubelka–Munk equation (Figure 2b).

The spectra of US50B and US100B present a pronounced band centered at 400 nm and a broad tail spanning until 650 nm. A comparison with the literature data allows for the assignment of the symmetric blue band to the surface plasmon resonance (SPR) of the metallic silver nanoparticles [12,60] adsorbed on the matrix; the data confirm that the reduction process driven by BH_4_^-^ in these two samples is very efficient and metal-nanostructures are formed. The spectra recorded from the US50C and US100C samples show a much broader and less intense band covering all Vis region. The limited intensities of the SPR band in the case of the citrate-treated samples suggest that the metallic nanoparticles have a smaller contribution compared to the US50B and US100B composites. For all four samples, a structured absorption tail has been detected, as evidenced by the first derivative of the absorption spectra (Inset Figure 2b); based on the literature data, this absorption can be attributed to Ag_3_PO_4_ nanostructures present on the surface of the matrix [48,61]. This observation indicates that in all samples, although with different efficiencies, silver phosphate depositions are formed.

**Figure 3:**
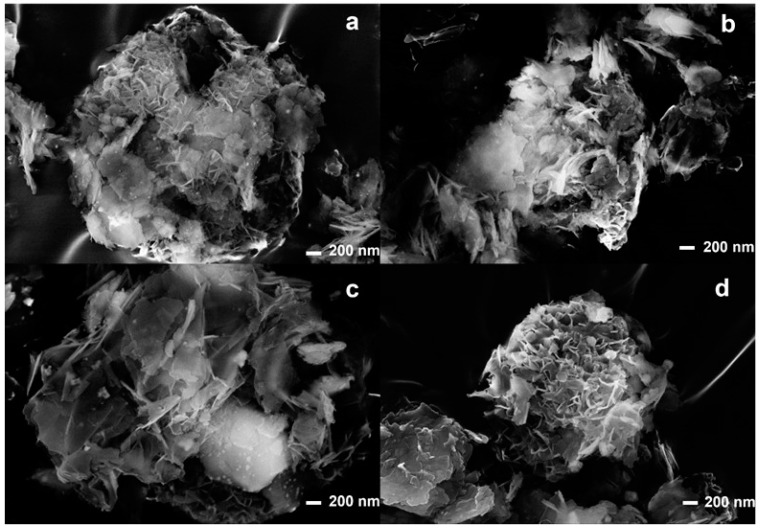
SEM analyses of US50B (**a**), US100B (**b**), US50C (**c**), and US100C (**d**).

Morphological characterizations were carried out using SEM-EDX and TEM analyses. Figure 3 shows the SEM analyses of the composites. It is possible to observe that in all composites, the lamellar meshwork structure remains unchanged after the ultrasound treatment.

**Figure 4:**
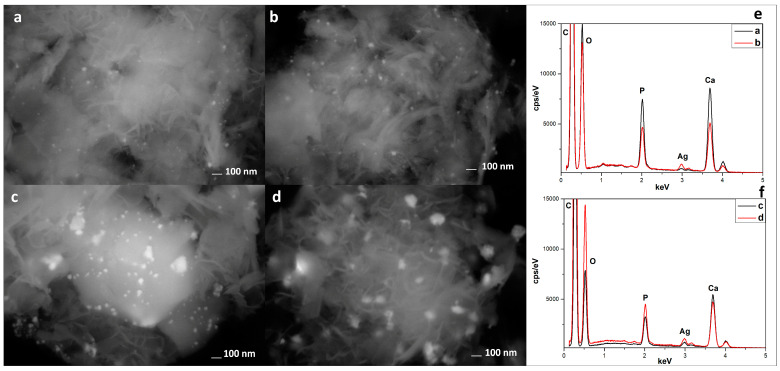
SEM analyses with a back-scattered detector of US50B (**a**), US100B (**b**), US50C (**c**), and US100C (**d**), and EDX spectra of US50B–US100B (**e**) and US50C–US100C (**f**).

In Figure 4, it is possible to observe the SEM analyses performed with a back-scattered detector (AsB) and the EDX analyses. The AsB detector highlights the presence and distribution of silver-based nanoparticles in all composites. In particular, in the sample US100C (Figure 4c), it is possible to observe a greater amount of silver nanoparticles, which is also confirmed by the EDX analyses (Figure 4f). 

**Figure 5:**
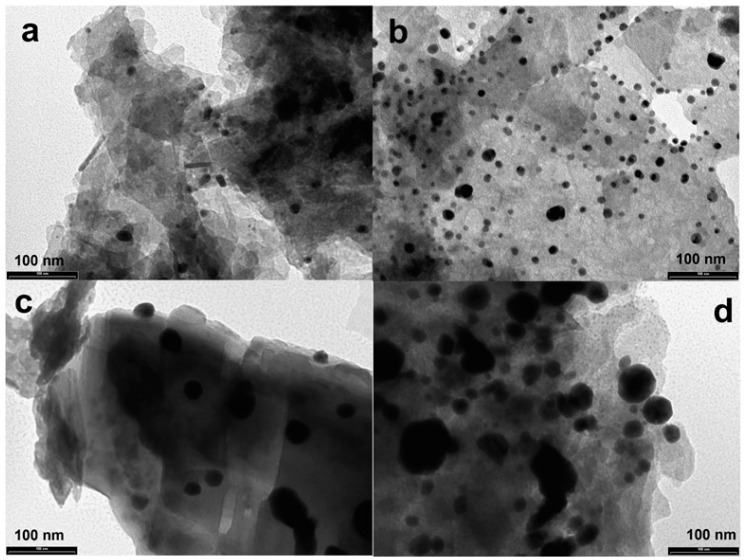
TEM images of US50B (**a**), US100B (**b**), US50C (**c**), and US100C (**d**).

To better highlight the shape and size, TEM analyses (Figure 5) were performed. The TEM images showed the good distribution on the OMP surface and the spherical shape of the silver-based nanoparticles; moreover, the average size is in agreement with that found by the Scherrer equation.

The experimental data showed that the use of ultrasound led to composites with a uniform loading of Ag nanoparticles due to a physical effect called acoustic cavitation [32]. As reported in the literature, the collapse of these cavitation bubbles generates extreme conditions, such as microstreaming, extreme shearing, and turbulence, which leads to the formation of nuclei at multiple locations, which, in turn, increases the number of nanoparticles that are finely loaded on the support [62].

For comparison and to highlight the effect of the ultrasound waves on the nanoparticle shape and size distribution, the synthesis of the composites of OMP and silver (named samples 50B and 50C) using the same reducing agents but without ultrasound irradiation has been performed, and the TEM images are shown in Figure S1. Of note is that the absence of ultrasound leads to silver aggregation.

**Figure 6:**
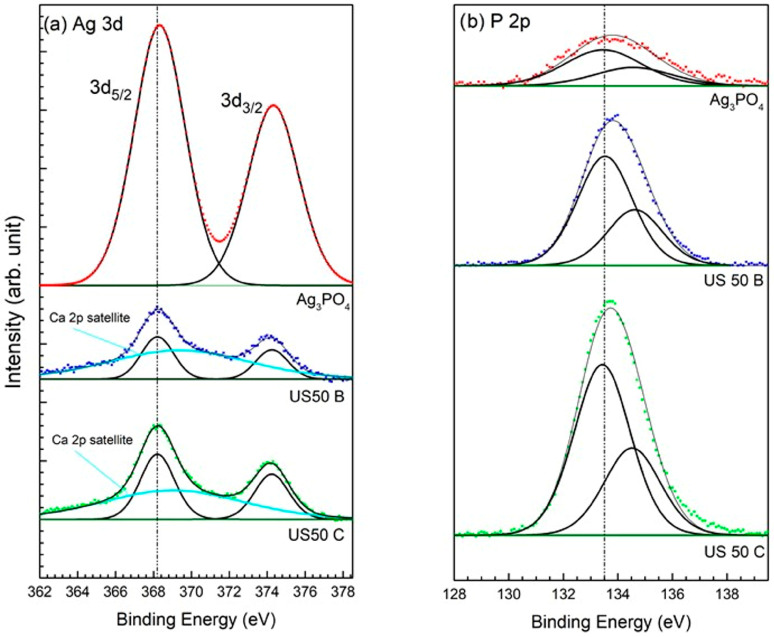
XPS spectra of the Ag 3d (**a**) and P 2p (**b**) regions of US50C, US50B, and the Ag_3_PO_4_ sample reference. The BE position of the main peaks is indicated by a dotted line.

Figure 6 presents the spectra of the main photoemission peaks Ag 3d and P 2p acquired for the OMP@silver composites and the Ag_3_PO_4_ reference sample. In all composites, the doublet of Ag 3d overlaps with the first shake-up satellite of the Ca 2p line [57], which was included in the peak fitting (Figure 6a). As we can see, the binding energy (BE) values of both elements are constant in all samples. Therefore, it was impossible to identify the oxidation state of Ag (0 or +1 in phosphate) only from the photoemission spectra. Therefore, Auger spectra of Ag M_4_N_5_N_5_ were acquired, and the modified Auger parameter α’ was calculated using their kinetic energy (KE). Then, from the comparison of the α’ values with a Wagner plot for Ag, also including the reference samples [57], it was possible to determine the oxidation state of Ag in the composites, and the results are presented in Table 3.

The discrepancy of the Ag oxidation states in comparison with the XRD results (see Table 2) can be explained by the different information depth, which is much higher in the XRD technique, whereas, in XPS, generally only a surface layer with a thickness of less than 10 nm is probed. In the case of the Auger peak Ag M_4_N_5_N_5_, the inelastic mean free path of electrons λ is about 1 nm [63], i.e., the information depth for the Auger parameter is only about 3λ = 3 nm. Intermediate values of α’ were observed on the surfaces of the two samples (USC50C and US100B), indicating a mixture of metallic and +1 (phosphate) states, whereas on the surfaces of the other two samples (US100C and US50B), only the metallic NPs of Ag were detected. The presence of Ag_3_PO_4_ in sample US100C, observed by XRD, can be explained by the lack of Ag reduction in the volume of this composite, where only metallic Ag NPs are present on the surface. Thus, it could be hypothesized that in the presence of a mild reducing citrate, first, Ag_3_PO_4_ nanoparticle formation takes place on the surface and bulk porous OMP [64], and then the presence of citrate provokes the reduction of silver that covers the superficial Ag_3_PO_4_ nanoparticles [65]. The reduction is total when a higher amount of citrate is used (US100C) and partial in the case of a lower amount (US50C). When NaBH_4_ is used, fast reduction of Ag^+^ takes place with the deposition of silver nanoparticles, both on the surface and in the bulk OMP.

### 3.2. Antibacterial Activity

For evaluating the composite antibacterial activity against *S. aureus* and *P. aeruginosa*, the MIC values were determined. MIC is the lowest concentration of an antimicrobial agent that inhibits the growth of a microorganism after 24 h of incubation. The MIC values of each composite and OMP are reported in Table 4 and are expressed as MIC referred to the composite (MIC) and MIC referred to the silver content in each composite (MIC_silver_), as reported in Table 1.

While the composites obtained by reduction with NaBH_4_ showed weak antimicrobial activity with MIC_silver_ values of 32.00 and 63.34 μg/mL against both of the tested microorganisms, the composites obtained in the presence of citrate showed good antibacterial activity with MIC_silver_ values between 3.87 and 7.97 μg/mL. These MIC_silver_ values were comparable to those of other silver@OMP composites obtained with different synthetic procedures, in which the presence of Ag_3_PO_4_ nanoparticles was detected [58]. These observations allow us to hypothesize that the presence of Ag_3_PO_4_ nanoparticles, in addition to those of metallic silver, improves the antimicrobial activity of the composites. In support of this hypothesis, an inverse correlation between the content of metallic silver in Ag_3_PO_4_@composites and bactericidal activity has previously been observed for hydroxyapatite-supported antibacterial Ag_3_PO_4_ nanoparticles [46]. Silver nanoparticles exert their antimicrobial activity through at least four different mechanisms, which are as follows: (i) adhesion of nanoparticles onto the cell wall surface and membrane; (ii) silver nanoparticle penetration into the cell and damage of the intracellular components and biomolecules; (iii) cytotoxicity and oxidative stress caused by free radicals and reactive oxygen species production; (iv) modulation of signal transduction pathways [66,67,68].

**Figure 7:**
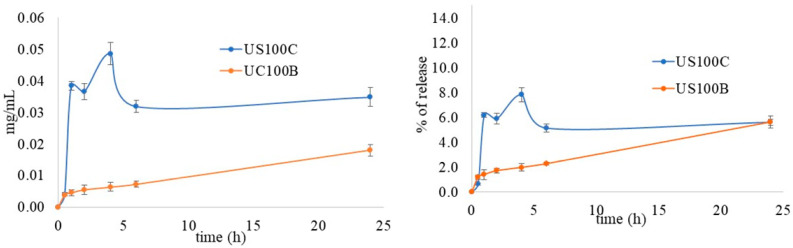
In vitro Ag^+^ release profile from the composites, expressed as Ag^+^ concentration (**left**) and % of release (**right**) as a function of time.

In order to explain the different antimicrobial activities between the composites obtained in the presence of citrate and those obtained in the presence of NaBH_4_ reduction, a silver in vitro release test was performed for the US100B and US100C composites, and the results are reported in Figure 7.

Silver ions were released slowly from both samples, but with some differences. The percentage of Ag^+^ released from US100B was lower than that from the sample prepared with citrate and did not reach a plateau after 24 h. The percentage of Ag^+^ released from US100C was higher and showed the presence of a burst effect, after which the release proceeded slowly, and, finally, a plateau was reached within 6h. This different silver release profile can be due to the nature of the nanoparticles in the composites. In fact, the characterization techniques proved the presence of metallic silver in US100B and both phosphate and metallic silver nanoparticles in US100C. The release of Ag^+^ from the metallic nanoparticles follows oxidative dissolution, which represents one more step to overcome before release, and this explains the very slow release. In US100C, the nanoparticles are in the form of both phosphate salt and metallic silver and an initial burst effect can be observed due to the silver in oxidized form in Ag_3_PO_4_ nanoparticles. This is in agreement with previously observed results [46,58]. Then, the silver profile from UC100C showed a maximum, which could correspond to a supersaturated solution where the silver concentration decreases successively [69].

The lower concentration of silver ions obtained from US100B could be the reason for its lower antibacterial activity. Moreover, OMP is characterized by high porosity, and its surface shows lamellar structures, whereas the deeper levels consist of smaller interconnected pores [64]. Thus, the small silver nanoparticles obtained by NaBH_4_ reduction can easily penetrate inside the lamellar and porous structures of the substrate, resulting in a lower number of nanoparticles on the outer surface and reducing the possibility of bacterial adhesion.

**Figure 8:**
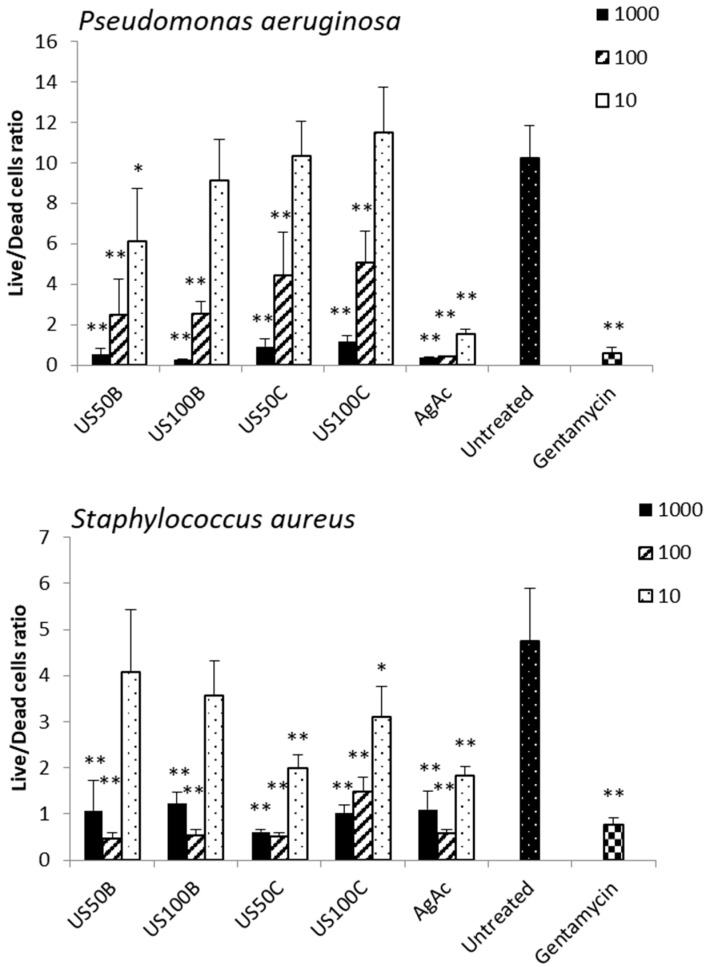
Live/dead ratio of *Pseudomonas aeruginosa* and *Staphylococcus aureus* biofilm cells after 24 h of incubation with 10, 100, and 1000 µg/L of the composites. Two independent experiments were performed in quadruplicate, and the data were reported as mean ± SD. A *t*-test was used for the statistical analysis of the raw data; * *p* < 0.05 and ** *p* < 0.01 (composite-treated or gentamycin-treated bacteria vs. untreated cells).

The results of the antibiofilm activity of the composites against *S. aureus* and *P. aeruginosa* are reported in Figure 8. All composites, both those obtained in the presence of NaBH_4_ and citrate, inhibited biofilm formation at the doses of 1000 and 100 μg/mL. Considering the different silver release profiles from the samples, these results suggest that the antibiofilm activity may be ascribed not only to the Ag^+^ release but also to different mechanisms. 

### 3.3. Cytotoxicity Evaluation

The composites were contacted with a monolayer of the HuDe and NCTC2544 cell lines in order to investigate their biocompatibility. The viability of the cell lines after 4 or 24 h of contact was evaluated, and the results are reported in Table 5 and compared to those obtained from the silver acetate.

The results are expressed as CC_50_, which is the concentration necessary to reduce the live cell number by 50% in comparison to the untreated controls. The CC_50_ values are reported as the means from two independent experiments performed in duplicate (Table 5).

The composites that were in contact with the NCTC2544 keratinocytes were less toxic than the silver acetate after 4 h, but the toxicity levels observed after 24 h were comparable to those obtained for AgAc. Conversely, the cytotoxicity of the composites observed towards the HuDe fibroblasts was comparable to that of the silver acetate at both 4 and 24 h of contact. The CC_50_ values for the keratinocytes of US50C and US100C after 4 h were higher than the MIC values against both bacteria, but this advantage disappeared after 24 h. These results allow us to confirm the narrow therapeutic window of silver nanoparticles [68]. However, it is interesting to underline that the US50C and US100C samples, which showed better antimicrobial activity, were not more cytotoxic than the others, and, thus, the presence of Ag_3_PO_4_ nanoparticles improves the antimicrobial activity without increasing the cytotoxicity.

## 4. Conclusions

In this paper, silver@hydroxyapatite functionalized CaCO_3_ composites were obtained using a sustainable ultrasound-assisted method. From the characterization, the presence of silver phosphate nanoparticles that were homogeneously distributed with the addition of metallic silver was revealed. For comparison, the ultrasound-assisted procedure was performed in the presence of the strong reducing agent NaBH_4_. In this case, smaller metallic silver nanoparticles were obtained.

These results confirm that the use of ultrasound favors the formation of spherical and homogeneously distributed nanoparticles, whose nature depends on the type of reducing agent. An antimicrobial activity evaluation revealed that the composites containing silver phosphate/metallic silver nanoparticles showed higher activity than those containing metallic nanoparticles. In conclusion, the proposed sustainable ultrasound-assisted procedure with citrate produces composites with good antimicrobial activity against *S. aureus* and *P. aeruginosa* without increasing the cytotoxicity.

## Figures and Tables

**Table 1:** Reaction conditions.

Sample	Ag(CH_3_COO) (M)	Reducing Agent (RA) (M)	Molar Ratio Ag/RA	Temperature (°C)
US50B	0.005	NaBH_4_ 0.06	1:10	25
US100B	0.010	NaBH_4_ 0.12	1:10	25
US50C	0.005	Sodium Citrate 0.014	1:2.3	85
US100C	0.010	Sodium Citrate 0.028	1:2.3	85

**Table 2:** Diameter of the silver or silver phosphate NPs obtained from the Scherrer equation and the silver content in each composite.

Sample	Silver Phase	NPs Diameter (nm)			Ag Content (*w*/*w*% ± 0.3%)
		2 Theta (°)	FWHM (°)	Calculated ^a^	
US50B	Ag	38.129	0.321	31	2.1
US100B	Ag	38.134	0.407	24	3.2
US50C	Ag	38.119	0.527	18	3.2
US100C	Ag_3_PO_4_	33.347	0.195	58	6.2
		36.642	0.173	69	

^a^ Calculated from the Scherrer equation: D = 0.9 × λ(δ × cosθ) (λ = incident wavelength: 1.54050 Å; δ = FWHM correct for instrumental broadening with LaB_6_ standard profile; θ = diffraction angle) applied on the (111) reflections of the cubic phase of Ag and on the (210) and (211) reflections of the cubic phase of Ag_3_PO_4_.

**Table 3:** The values of BE and KE for the Ag 3d_5/2_ and Ag M_4_N_5_N_5_ peaks, the Auger parameter α’, and the Ag oxidation state in the OMP@silver composites.

Sample	Ag 3d_5/2_ BE, eV	Ag M_4_N_5_N_5_ KE, eV	α′, eV	Ag Oxidation State
US50C	368.2	356.7	724.9	+1/metallic
US100C	368.2	357.5	725.7	Metallic
US50B	368.2	357.5	725.7	Metallic
US100B	368.2	357.2	725.4	+1/metallic

**Table 4:** MIC and MIC_silver_ values of the composites towards *S. aureus* and *P. aeruginosa* strains.

Sample	MIC (μg/mL) *S. aureus*	MIC_silver_ (μg/mL) *S. aureus*	MIC (μg/mL) *P. aeruginosa*	MIC_silver_ (μg/mL) *P. aeruginosa*
OMP	>1000	-	>1000	-
US100C	62.5	3.87	125	7.75
US50C	125	3.99	250	7.97
US100B	1000	32.00	1000	32.00
US50B	1000	63.34	1000	63.34
AgAc	3.9	2.52	3.9	2.52
Gentamicin	0.24	-	0.97	-

The results are representative of two independent experiments with overlapping values.

**Table 5:** Cytotoxicity of the composites against the HuDe and NCTC2544 cell lines.

CC50 (µg/mL)	NCTC2544		HuDe	
	4 h	24 h	4 h	24 h
US50C	253.7 ± 11.4	101.9 ± 2.8	89.6 ± 3.3	56.9 ± 4.0
US100C	421.5 ± 36.4	86.7 ± 6.4	92.6 ± 9.4	29.3 ± 3.5
US50B	259.7 ± 22.3	119.6 ± 12.2	101.7 ± 3.0	38.0 ± 3.4
US 100B	315.9 ± 24.4	149.6 ± 11.6	92.0 ± 10.7	41.1 ± 5.8
AgAc	130.3 ± 8.3	171.7 ± 7.8	108.9 ± 10.5	44.7 ± 7.1

Data represent the mean ± SD of two independent experiments.

## Data Availability

Not applicable.

## Supplementary Materials

The following supporting information can be downloaded at: https://www.mdpi.com/article/10.3390/ma16041338/s1, Figure S1: TEM images of 50C (a) and 50B (b).
