# Supplementary material for: Ag/Ag3PO4 Nanoparticle-Decorated Hydroxyapatite Functionalized Calcium Carbonate: Ultrasound-Assisted Sustainable Synthesis, Characterization, and Antimicrobial Activity
Source: Materials (Basel). 2023 Feb 4;16(4):1338. doi: 10.3390/ma16041338 (PMC9962710; doi:10.3390/ma16041338)
Supplement: Supplementary file 1 [file materials-16-01338-s001.zip › materials-2161260-supplementary.pdf]

Supplementary materials

Ag/Ag_3PO_4 Nanoparticle-Decorated Hydroxyapatite Functionalized Calcium Carbonate: Ultrasound-Assisted Sustainable Synthesis, Characterization, and Antimicrobial Activity

Alessandro Di Michele [1], Morena Nocchetti [2,*], Donatella Pietrella [3], Loredana Latterini [4], Giulia Quaglia [4], Ilaria Mattu [2], Giuseppina Padeletti [5], Saulius Kaciulis [5], Eleonora Bolli [5] and Valeria Ambrogi [2,*]

[1] Dipartimento di Fisica e Geologia, University of Perugia, Via Alessandro Pascoli, 06123 Perugia, Italy
[2] Dipartimento di Scienze Farmaceutiche, University of Perugia, Via del Liceo 1, 06123 Perugia, Italy
[3] Dipartimento di Medicina e Chirurgia, University of Perugia, Via Gambuli, 1, 06132 Perugia, Italy
[4] Nano4Light Lab, Dipartimento di Chimica, Biologia e Biotecnologie, University of Perugia, Via Elce di Sotto 8, 06123 Perugia, Italy
[5] Institute for the Study of Nanostructured Materials, ISMN-CNR, Via Salaria Km 29,300, 00015 Monterotondo, Italy
* Correspondence: morena.nocchetti@unipg.it (M.N.); valeria.ambrogi@unipg.it (V.A.)

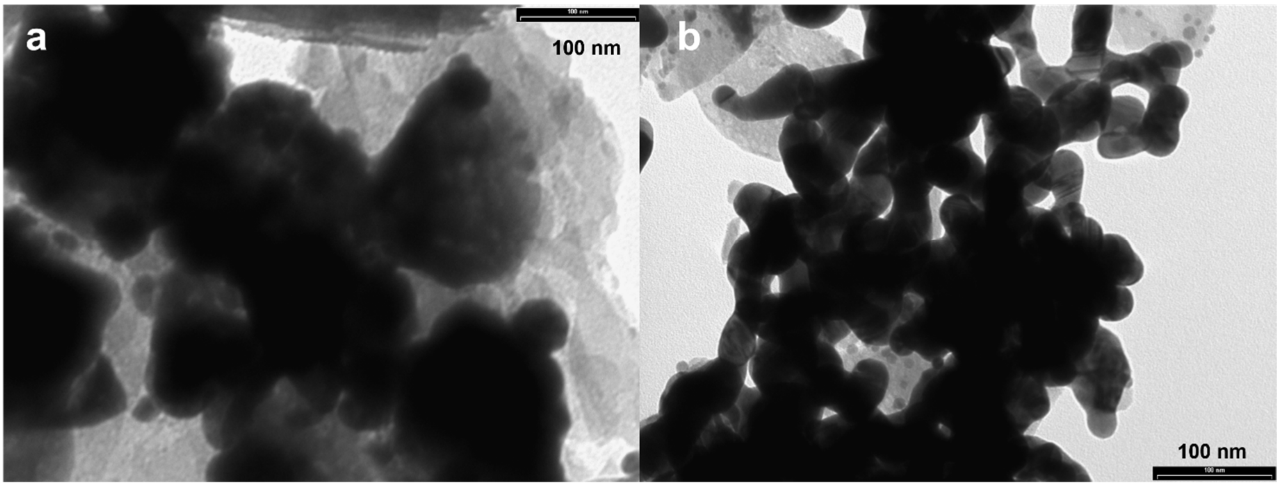

Figure S1: TEM images of 50C (a) and 50B (b).
